# Gaze strategy and sense of ownership in learning prosthetic control: a comparative study using wearable eye tracking

**DOI:** 10.1186/s12984-025-01860-0

**Published:** 2025-12-27

**Authors:** Manabu Yoshimura, Hiroshi Kurumadani, Shota Date, Junya Hirata, Tomotaka Ito, Katsutoshi Senoo, Kozo Hanayama, Toru Sunagawa

**Affiliations:** 1 Kawasaki University of Medical Welfare, 288 Matsushima, Kurashiki, 701-0192 Okayama Japan; 2 Faculty of Medicine, Nagoya City University, 1 Kawasumi, Mizuho-cho, Mizuho-ku, Nagoya, 467-8601 Aichi Japan; 3 Graduate School of Biomedical and Health Sciences, Hiroshima University, 1-2-3 Kasumi, Minami-ku, Hiroshima, 734-8551 Japan; 4 Department of Rehabilitation Medicine, Kawasaki Medical School, 577 Matsushima, Kurashiki, 701-0192 Okayama Japan

**Keywords:** Prosthetic control, Eye tracking, Body-powered prosthesis, Myoelectric prosthesis, Sense of ownership, Cognitive load, Motor learning

## Abstract

**Background:**

Prosthetic control requires not only motor execution but also the development of adaptive visual strategies. Myoelectric prostheses provide limited sensory feedback and therefore rely more heavily on visual monitoring. However, learning-related changes in gaze behavior—including fixation patterns and physiological indices such as blink rate—remain underexplored. This study aimed to investigate how gaze behavior changes and the sense of ownership change during the learning of body-powered and myoelectric prosthetic control, and how these effects differ depending on hand dominance.

**Methods:**

Thirty-six healthy adults (18 males and 18 females) were randomly assigned to four groups: body-powered prosthesis with dominant hand, body-powered with non-dominant hand, myoelectric with dominant hand, and myoelectric with non-dominant hand. Participants performed a simulated prosthetic control task (Coin Task from the Southampton Hand Assessment Procedure) before and after training. Gaze behavior was recorded at 50 Hz using Tobii Pro Glasses 3. Primary outcomes included gaze fixation percentage, blink rate, task completion time, and self-reported sense of ownership. To enhance the ecological validity of the findings, two participants with upper limb amputation who regularly used prostheses also completed the task using their own devices.

**Results:**

All groups demonstrated reduced task completion time and increased sense of ownership following training (*p* < .01). The body-powered groups exhibited increased fixation on the target (jar) during the lift phase, suggesting predictive gaze use. In contrast, the myoelectric groups maintained gaze on the hand or object, indicating compensatory strategies. Blink rate did not change significantly. The participants with upper limb amputation also showed high jar fixation and a strong sense of ownership. The participant using a body-powered prosthesis demonstrated a gaze pattern consistent with the predictive gaze observed in able-bodied users of the body-powered simulator, whereas the participants using myoelectric prostheses showed high jar fixation that differed from the hand-centered fixation typically seen in able-bodied myoelectric users. No significant effects of hand dominance were observed in any of the gaze or blink rate measures.

**Conclusions:**

Short-term prosthetic training improved task performance and increased the sense of ownership across all groups. Body-powered and myoelectric prosthesis control showed characteristic differences in gaze strategies; however, these differences did not emerge as significant main effects of prosthesis type. Hand dominance also had no significant effects on gaze or blink-related measures. The findings suggest that training-induced changes, rather than prosthesis type or side of control, primarily shaped gaze behavior during early prosthetic learning. Participants with upper limb amputation demonstrated efficient gaze allocation and a strong sense of ownership, indicating possible adaptations associated with long-term prosthesis use.

## Background

Prosthetic devices are used to replace lost upper limb function and are typically classified into body-powered prostheses (BP), myoelectric prostheses (MP), cosmetic prostheses, and work prostheses. Among these, BP and MP—which allow voluntary control of a terminal device such as a hook or hand—are considered the most practical options for activities of daily living.

BP are internally powered devices operated through cable systems actuated by shoulder and scapular movements. Because control requires the user’s own body motion, BP users can rely on proprioceptive feedback, which may promote the sense of ownership [1]. However, acquiring proficiency with BP demands repetitive practice and motor adaptation. In contrast, MP are externally powered prostheses that control terminal device movement using electromyographic (EMG) signals derived from residual muscles [2]. Although MP enable relatively intuitive control, they provide limited sensory feedback, resulting in an increased reliance on visual information during operation.

Previous studies have shown that myoelectric prosthesis users rely heavily on visual information, as evidenced by prolonged gaze fixations on the terminal device during control tasks [3, 4]. This reliance on visual input may elevate cognitive load, reduce task efficiency, and contribute to user fatigue [5]. Gaze behavior serves as a sensitive marker of motor learning: novices tend to fixate on the prosthetic hand, whereas experienced users employ predictive gaze strategies that anticipate upcoming actions [6, 7].

Blink rate, another gaze-derived physiological index, has been shown to decrease with increased cognitive or mental workload [8]. In cognitively demanding tasks such as prosthetic control, blink rate may serve as a non-invasive and real-time indicator of user workload. Therefore, the simultaneous evaluation of gaze fixation patterns and blink rate may offer a multidimensional understanding of attentional strategies and cognitive demands during prosthetic use [5].

Furthermore, the development of a sense of ownership—defined as the subjective experience that the prosthetic limb is part of one’s own body—is a critical factor in prosthetic control. The sense of ownership, a key component of prosthetic embodiment, is influenced by factors such as sensorimotor congruence [9], consistency in control, and the quality of feedback. A heightened sense of ownership is clinically important, as it is associated with improved user motivation and long-term prosthesis acceptance [9].

Furthermore, hand dominance has been reported to influence visuomotor control, motor learning, and cortical activity patterns during tool use and reaching movements [10]. Therefore, in prosthetic control learning, it is plausible that gaze behavior and the sense of ownership may differ depending on whether the dominant or non-dominant hand is used.

Despite the clinical relevance of these constructs, few studies have investigated how prosthesis type (BP vs. MP) and hand dominance (dominant vs. non-dominant hand) influence gaze behavior and the sense of ownership during the learning process. In particular, how gaze behavior changes from baseline to post-training—and how these changes interact with prosthesis type and ownership—remains poorly understood.

Hand dominance was included as a factor because previous studies suggest that non-dominant hand use may require greater attentional resources during novel motor tasks, potentially influencing gaze allocation [10]. However, due to insufficient evidence to predict its specific effects in prosthetic control, hand dominance was treated as an exploratory factor rather than a hypothesis-driven variable.

This study aimed to quantitatively assess gaze behavior using wearable eye tracking and to compare the evolution of gaze fixations during training with body-powered and myoelectric prostheses. In addition, we examined how prosthesis type and hand dominance affect task performance, cognitive workload (assessed through blink rate), and the subjective sense of ownership.

Overall, this study represents a preliminary step toward understanding the early stage of prosthetic learning and how hand dominance influences gaze behavior and the sense of ownership.

Based on the differing sensory feedback and motor control mechanisms between prosthesis types, we formulated the following hypotheses:

1. Users of BP would tend to develop gaze strategies that increasingly anticipate task-relevant targets, as BP systems provide proprioceptive feedback and direct mechanical linkage to body movement.
2. Users of MP might rely more consistently on visual monitoring of the prosthetic hand, given the reduced sensory feedback and relatively indirect control interface.
3. Regardless of prosthesis type or hand dominance, repeated training was expected to support the development of a stronger sense of ownership toward the prosthetic limb, facilitated by consistent sensorimotor experience and task engagement.

## Methods

### Participants

Thirty-six right-handed healthy adults (18 males and 18 females; mean age = 23.1 years, SD = 1.9), with no prior experience in prosthetic control, participated in the study (Table 1). All participants provided written informed consent after receiving an explanation of the study’s purpose and procedures. The study was approved by the Ethics Committee of Kawasaki University of Medical Welfare (Approval No. 24 − 018). The sample size was determined using G*Power (ver. 3.1) with an effect size of 0.4, α = 0.05, and power = 0.8, yielding a minimum required sample of 36 [11].

**Table 1:** Participants demographic data

Group	*N*	Sex (M/F)	Age (years) Mean (SD)	Edinburgh Inventory score mean (SD)
Domi-BP	9	5 / 4	21.89 (1.10)	93% (2.3)
Non-BP	9	4 / 5	22.56 (1.64)	98% (6.6)
Domi-MP	9	4 / 5	24.67 (4.11)	98% (6.6)
Non-MP	9	5 / 4	23.22 (1.40)	98% (4.8)

Mean (SD), SD = standard deviation

This table presents the participants’ demographic characteristics, including sex, age, and Edinburgh Handedness Inventory score

No significant differences were observed between groups in age or Edinburgh Handedness Inventory score

### Group assignment

**Fig. 1:**
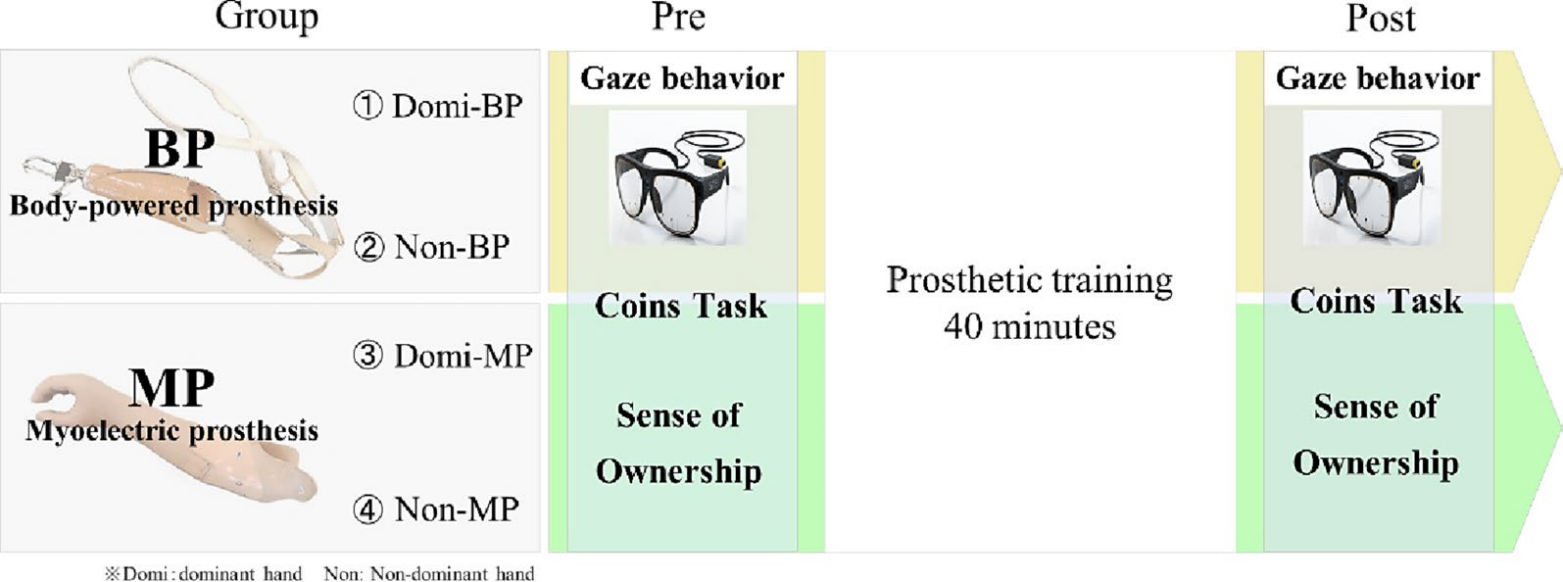
Experimental protocol and group assignment. Participants were randomly assigned to one of four groups based on prosthesis type (BP: body-powered; MP: myoelectric) and controlling hand (dominant or non-dominant). All participants followed a standardized protocol that included baseline measurement (Pre), a 40-minute prosthetic training session, and post-intervention measurement (Post). Assessments included gaze behavior, task performance (Coins Task), and sense of ownership

Participants were randomly assigned to one of four groups (*n* = 9 per group) based on two factors: prosthesis type (BP or MP) and controlling hand (dominant or non-dominant) (Fig. 1):

1. Domi-BP group: BP controlled with the dominant hand.
2. Non-BP group: BP controlled with the non-dominant hand.
3. Domi-MP group: MP controlled with the dominant hand.
4. Non-MP group: MP controlled with the non-dominant hand.

**Fig. 2:**
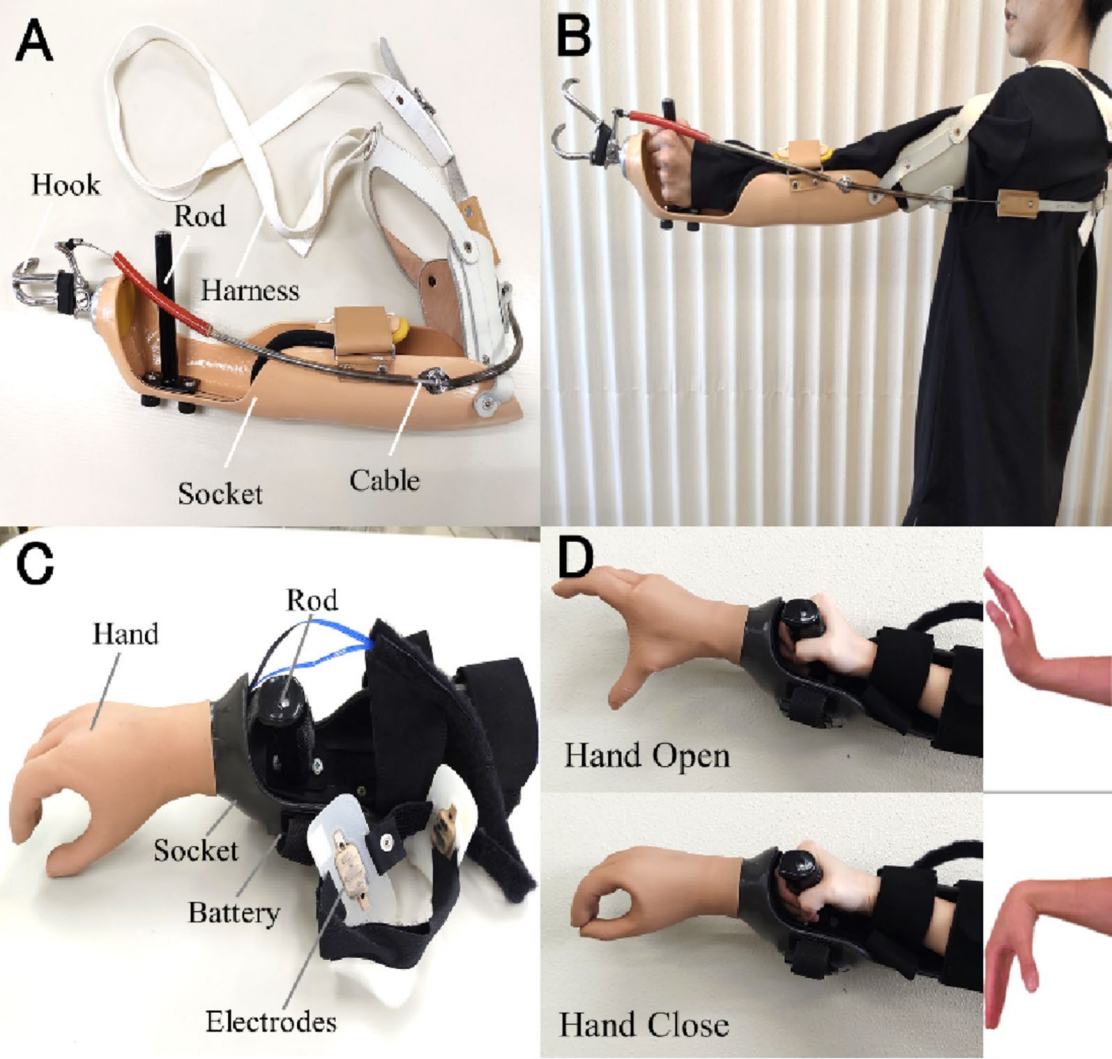
Simulated prosthetic devices used in the study. (A) The BP prosthetic simulator consists of a hand hook, rod, socket, cable, and harness. (B) The simulator is a voluntary-opening BP prosthesis. The hook opens with left shoulder flexion and scapular abduction, and closes with left shoulder extension and scapular adduction. (C) The MP simulator consists of a hand, socket, rod, electrodes, and battery. (D) The user’s hand opened during isometric contraction of the radial carpal extensor muscles and closed during contraction of the ulnar carpal flexor muscles. The figure illustrates isotonic contraction to aid understanding of hand manipulation

Participants were fitted with the assigned prosthesis and performed tasks under standardized procedures and environmental conditions.

### Simulated prostheses

Simulated prosthetic devices were used to mimic the weight and structure of actual prostheses, enabling consistent and safe operation by inexperienced users.

### Simulated body-powered prosthesis

The simulated BP used a cable system actuated by scapular abduction and shoulder flexion to open a hook-terminal device, replicating a conventional BP [12]. The design and mechanism are shown in Fig. 2AB. It consisted of a hook, socket, rod, cable, and harness and was configured as a voluntary-opening BP. Control was executed via a figure-eight harness wrapped around the contralateral shoulder. A trained occupational therapist adjusted the cable tension and hook orientation to ensure optimal fit and functionality.

### Simulated myoelectric prosthesis

The simulated MP employed surface electrodes placed on the forearm to detect EMG signals. When the signals exceeded a preset threshold, a motor-driven prosthetic hand opened or closed accordingly [13]. The device structure is shown in Fig. 2CD. The system included a hand, socket, rod, battery, and electrodes. We used the MyoHand VariPlus Speed (Ottobock), which adjusted hand movement speed proportionally to EMG signal strength [14]. A trained occupational therapist placed the electrodes over the extensor carpi radialis and flexor carpi ulnaris muscles. The hand opened in response to isometric contraction of the extensor and closed with contraction of the flexor.

### Prosthetic control task

**Fig. 3:**
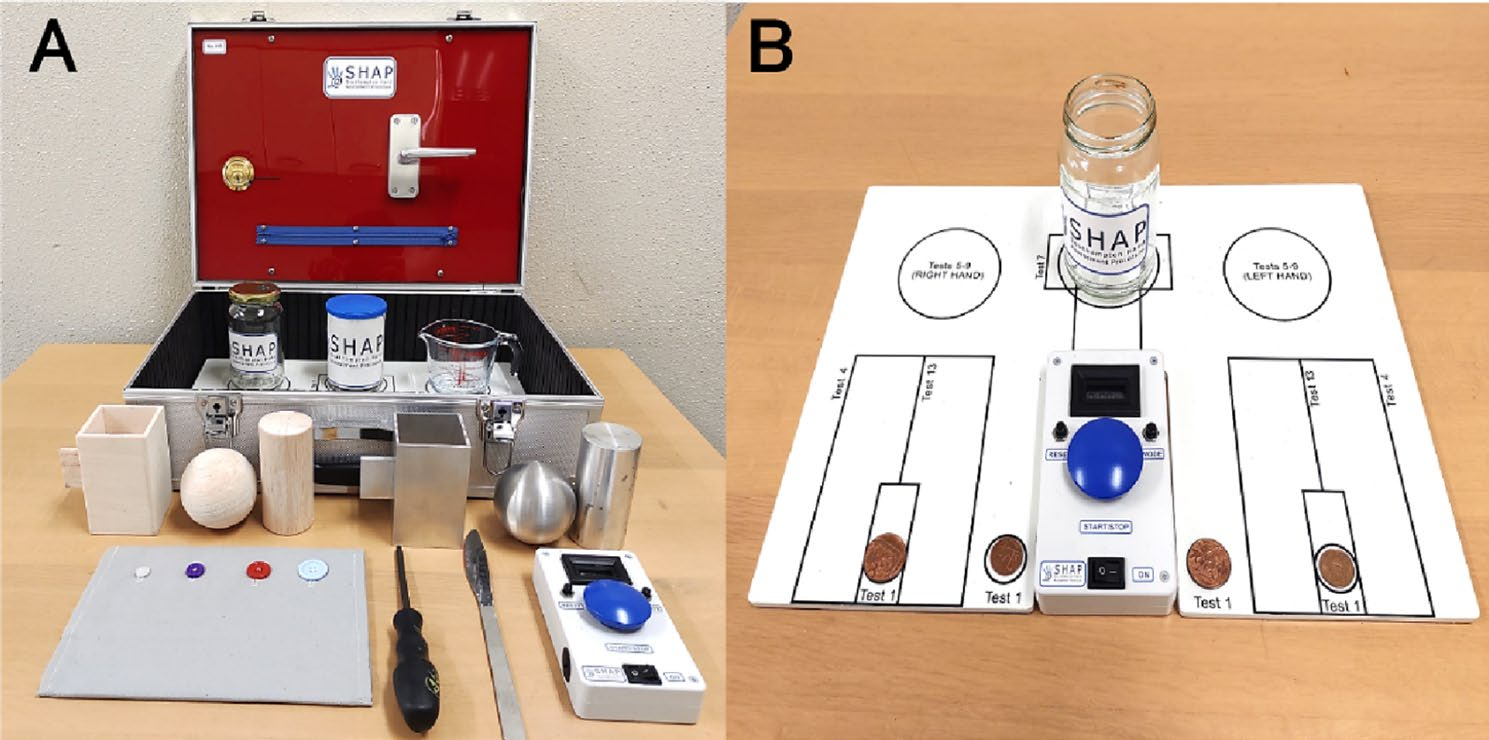
Experimental setup for the Coins Task. (A) Task apparatus including the coin, jar, and prosthesis. (B) Participant performing the task using the simulated prosthesis

The Coins Task from the SHAP was used to assess prosthetic performance [15]. Participants picked up coins one by one and placed them into a jar. A depiction of the task setup and procedure is provided in Fig. 3. Task duration was measured using a blue-timer button at the start and finish. This task simulated everyday object handling, requiring coordinated visual search, motor control, and grasping precision, thereby serving as an effective probe for detecting changes in gaze strategy.

### Eye tracking

**Fig. 4:**
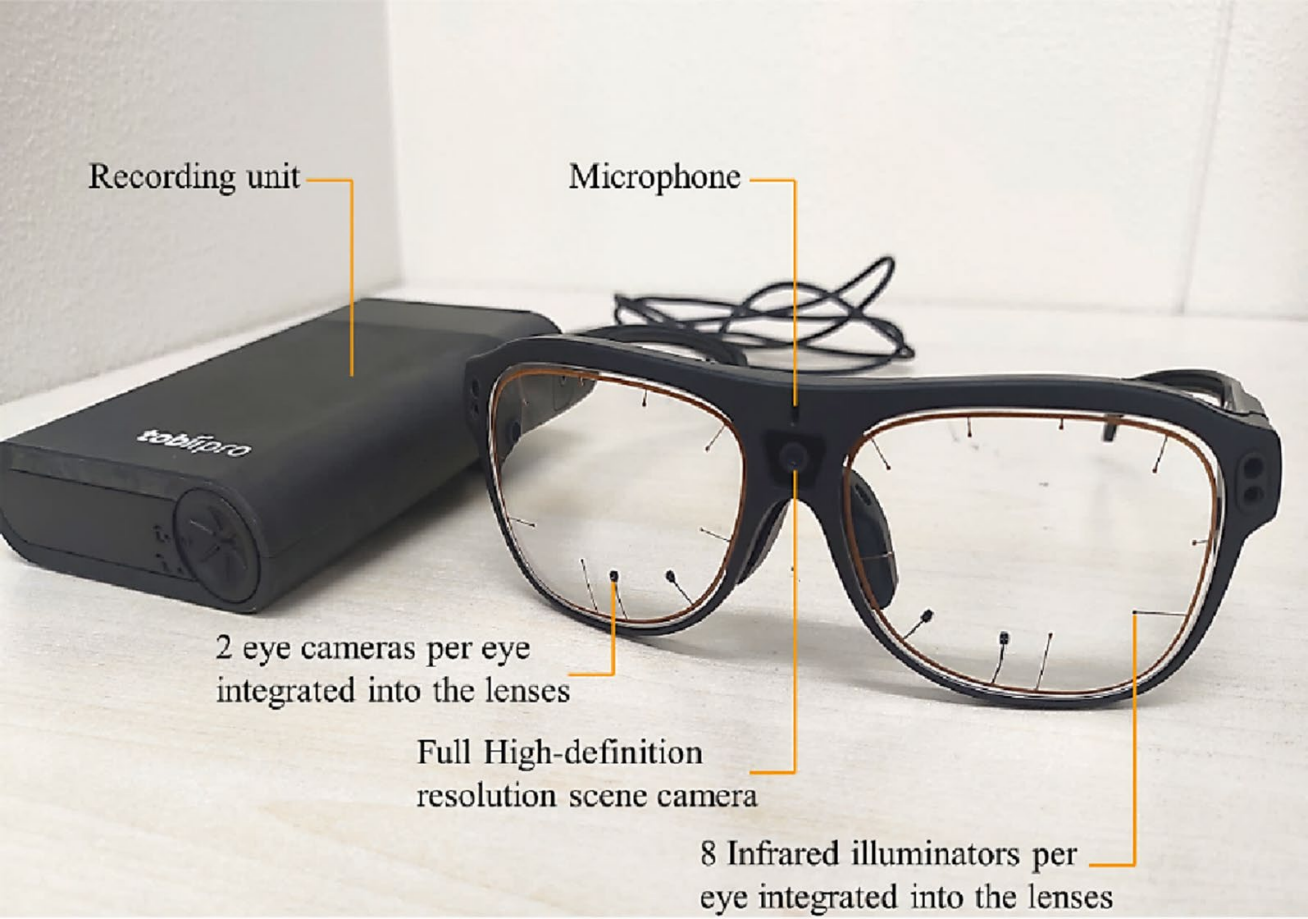
Tobii Pro Glasses 3 eye-tracking system used for gaze analysis. Overview of the Tobii Pro Glasses 3 eye-tracking system used to record gaze behavior during prosthetic task performance. The system includes a full high-definition scene camera, two eye cameras per eye integrated into the lenses, and eight infrared illuminators per eye to detect pupil position. A recording unit and built-in microphone captured synchronized gaze, audio, and behavioral data

Eye movements were recorded using Tobii Pro Glasses 3 (Tobii AB, Danderyd, Sweden) [16] (Fig. 4), a wearable eye tracker that allows natural behavior in free-viewing environments. The sampling rate was set at 50 Hz, and data were analyzed using Tobii Pro Lab software (Tobii AB, Danderyd, Sweden). Fixation events were automatically extracted using the Identification by Velocity Threshold algorithm with a default velocity threshold of 30°/s in Tobii Pro Lab. Fixations were defined as clusters of gaze points that remained within a spatial radius of 1° of visual angle for at least 60 ms.

Before analysis, gaze data were filtered using Tobii’s built-in noise reduction filter, and blinks or missing data segments were excluded from fixation calculations. Trials with tracking loss exceeding 15% were excluded from analysis.

### Definition of areas of interest (AOIs)

To evaluate gaze allocation toward task-relevant objects, Areas of Interest (AOIs) were manually defined in Tobii Pro Lab by visually checking each frame. The coin AOI was delineated along the coin’s contour with minimal margins, while the hand AOI was restricted to the prosthetic hook tip or hand region only, minimizing overlap as much as possible. In instances such as the grasping phase, where the hand and coin partially overlapped, we followed the default setting of Tobii Pro Lab, classifying fixations within the overlapping area as belonging to both AOIs.

### Percentage of fixation (% fixation)

**Fig. 5:**
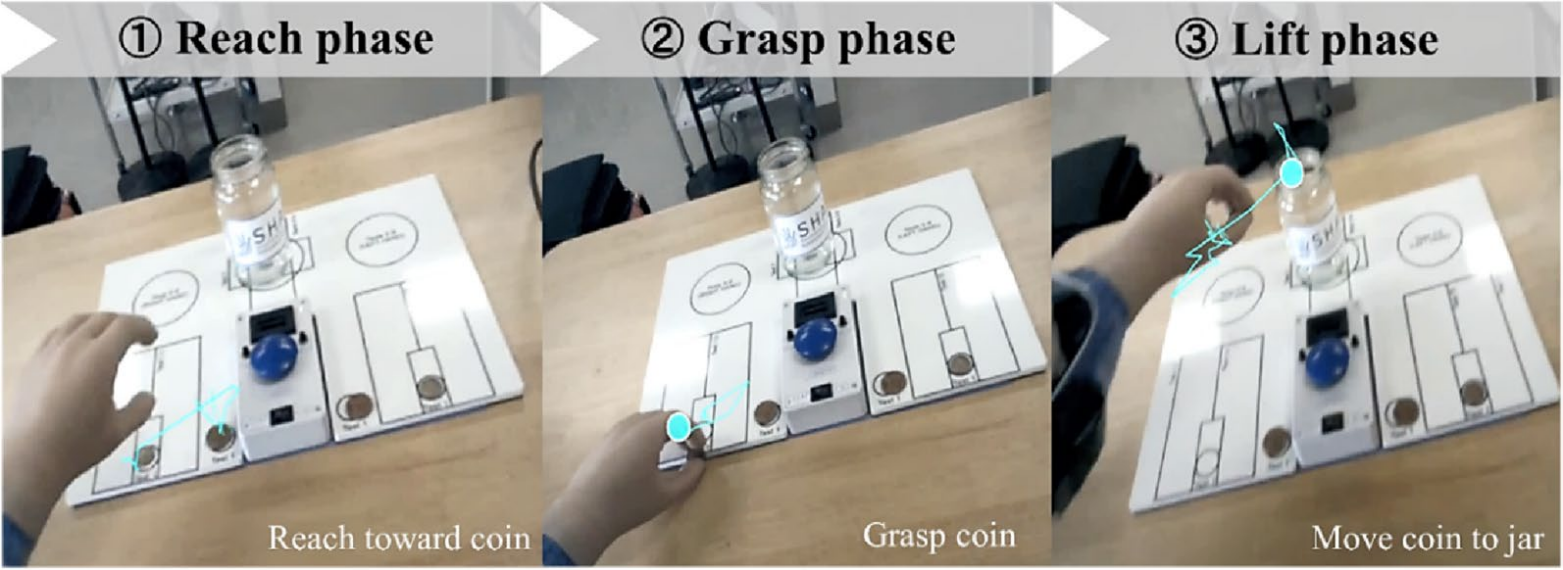
Definition of task phases. The Coins Task was divided into three phases: (1) Reach phase—reaching toward the coin; (2) Grasp phase—grasping the coin; (3) Lift phase—lifting and moving the coin to the jar

**Fig. 6:**
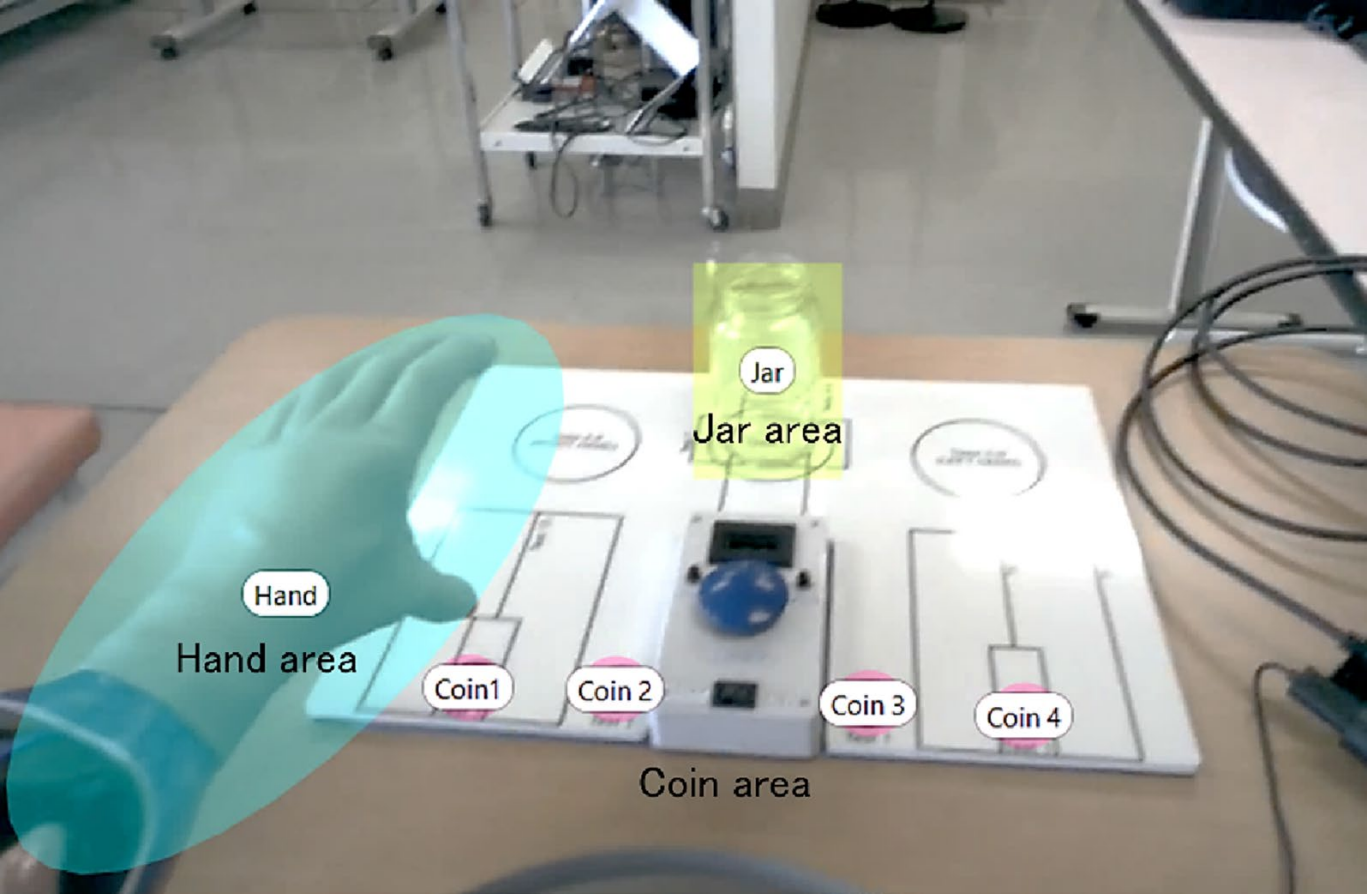
Definition of AOIs. Gaze fixation was analyzed within three AOIs: (1) hand area, (2) coin area, and (3) jar area

Time of Interest (TOI) phases were defined as Reach (approach to coin), Grasp, and Lift (move to jar) (Fig. 5). Areas of Interest (AOIs) included the prosthetic hand, coin, and jar (Fig. 6). % Fixation was calculated by dividing the total fixation time on each AOI by the total gaze duration within the corresponding TOI. This metric quantified the allocation of visual attention and reflected attentional focus during prosthetic control. Higher fixation percentages indicated more concentrated attention on a given object [4, 17]. By comparing % Fixation across TOIs and AOIs, we identified attentional priorities and visualized how visual attention was distributed throughout task execution.

### Blink rate

Blinks were identified as missing pupil data lasting between 30 ms and 3000 ms [18]. Blink frequency was computed as the number of blinks per minute and compared before and after training. Because blinking tends to decrease with increased cognitive or mental load [8], this index was used alongside fixation data to monitor cognitive demands. Blink analysis was performed using MATLAB (MathWorks, Natick, MA, USA) and custom scripts applied to pupil data exported from Tobii Pro Lab.

To enhance reliability, only binocular loss of pupil data was considered a valid blink. Transient data loss under 30 ms was excluded as noise. All recordings were visually inspected for artifacts such as goggle slippage or lighting-induced signal dropouts. Trials with more than 15% missing data were excluded from analysis.

### Additional evaluation measures

To comprehensively assess learning outcomes and psychological effects, the following measures were also evaluated:

1. Task Completion Time: The time (in seconds) required to complete the Coins Task before and after training was recorded as an indicator of prosthetic skill acquisition. As a quantitative index of proficiency in prosthetic control, the rate of change after training was calculated relative to the pre-training value (set at 100%) and used for analysis.
2. Sense of Ownership: Subjective ownership was assessed using a 7-point Likert scale adapted from Kalckert et al. [19], based on the statement: “I felt as if I was controlling the prosthesis as if it were my own hand.” Participants rated their agreement from − 3 (strongly disagree) to + 3 (strongly agree), and this score was used as the ownership index.

This item has been widely used in studies on prosthesis embodiment and the rubber hand illusion, and is recognized as a valid measure of subjective ownership [19].

### Protocol

All participants followed the same protocol (Fig. 1). Each began with a 5-minute explanation and demonstration of the assigned prosthesis, followed by practice trials to become familiar with the device. Baseline measurements (Pre) were recorded during two Coins Task trials, capturing gaze behavior, task completion time, and ownership ratings.

Next, participants underwent 40 min of prosthetic training, repeatedly practicing the Coins Task with emphasis on timing, alignment, and grasp accuracy. Training was conducted under the supervision of a trained occupational therapist, who provided standardized verbal guidance and corrective feedback when necessary. Each training trial was separated by approximately 1-minute intervals to reduce fatigue, and rest breaks were allowed as needed.

To ensure consistency across participants, both the order of the Coins Task and the spatial arrangement of the coins were fixed throughout all sessions. All sessions were conducted in a quiet, well-lit environment, and all assessments were performed by the same examiner to ensure procedural consistency.

After training, participants completed two additional Post trials under identical conditions. Post measurements included gaze data, blink rate, task completion time, and ownership ratings. These trials enabled the evaluation of training-induced changes in attentional strategy, motor efficiency, and sense of ownership.

The present training was designed to evaluate short-term task familiarization (pre–post) rather than long-term skill acquisition. Future studies should employ longitudinal protocols to examine progressive learning and adaptation.

### Statistical analysis

All statistical analyses were performed using IBM SPSS Statistics version 26 (IBM Corp.). Normality was tested using the Shapiro–Wilk test. Parametric tests, including paired t-tests and two-way repeated-measures ANOVA, were applied to normally distributed variables.

The primary models examined interactions between Session (Pre vs. Post) and Group (Domi-BP, Non-BP, Domi-MP, Non-MP), as well as between Session and AOI (Hand, Coin, Jar). Gaze data were analyzed separately for each phase (Reach, Grasp, Lift).

Blink rate and ownership ratings were compared across groups and sessions, with post hoc testing conducted using Holm-corrected t-tests where appropriate. Task completion time change rates were analyzed in the same manner. A significance level of *p* < .05 was used. Effect sizes (Cohen’s d or partial η²) were calculated to assess the magnitude of observed differences.

## Results

### Changes in task completion time (Fig. 7A)

**Fig. 7:**
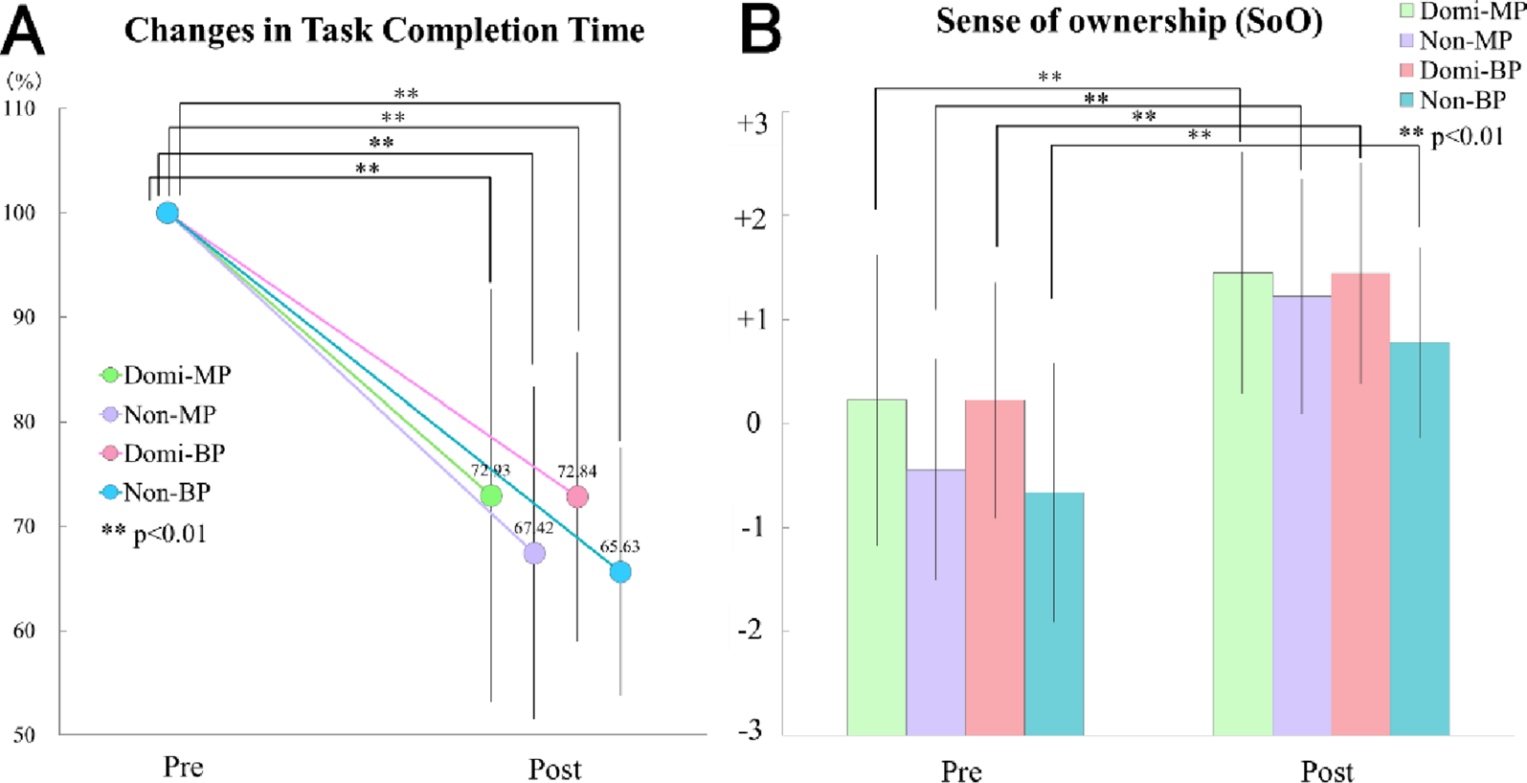
Changes in task completion time and sense of ownership. (A) Task completion time significantly decreased in all groups after training. (B) Sense of ownership scores significantly increased in all groups. *p* < .01. Error bars represent mean ± standard deviation (SD)

Task completion time significantly decreased after training across all groups (main effect of Session: F(1,8) = 107.625, *p* < .01, η² = 0.757), as shown in Fig. 7A. The main effect of Group and the Session × Group interaction were not significant (*p* > .05), indicating that the magnitude of improvement was comparable among groups. Post hoc pairwise comparisons between groups at the Post session using Holm-corrected t-tests also confirmed no significant differences.

### Changes in sense of ownership (Fig. 7B)

As depicted in Fig. 7B, all four groups exhibited significant increases in the sense of ownership following prosthesis use (main effect of Session: F(1,8) = 136.986, *p* < .01, η² = 0.390). No significant main effect of Group or Session × Group interaction was observed (*p* > .05). Holm-corrected t-tests conducted at the Post session confirmed no significant group differences.

### Gaze behavior: changes in fixation percentage (Fig. 8; Table 2)

**Fig. 8:**
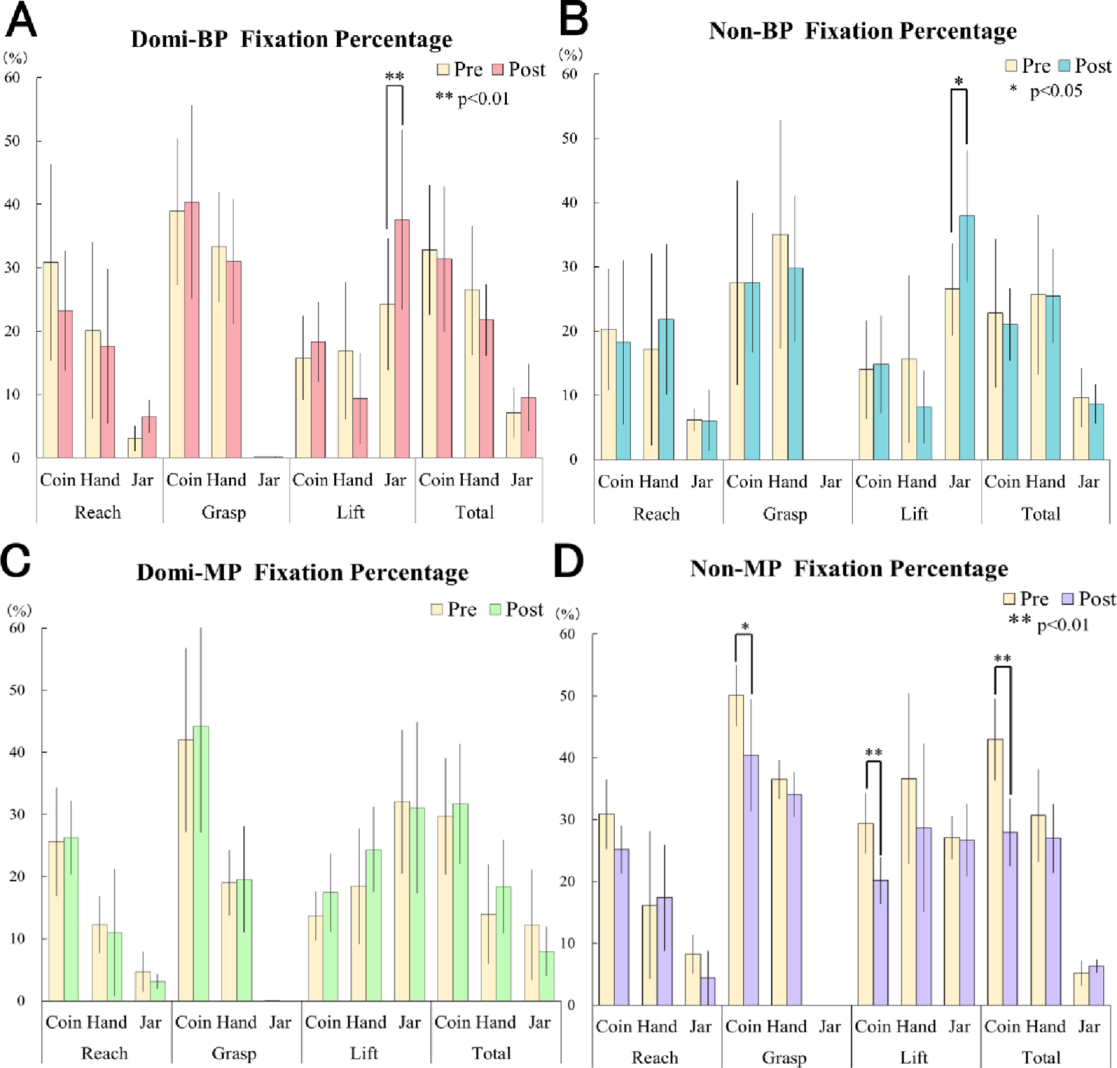
Gaze fixation percentage in BP and MP groups. (A) Domi-BP group: fixation on the jar significantly increased during the Lift phase after training (*p* < .01). (B) Non-BP group: a similar increase in jar fixation was observed (*p* < .05). (C) Domi-MP group: no significant increase in jar fixation was observed; gaze remained primarily focused on the hand and coin. (D) Non-MP group: similarly, no significant increase in jar fixation was found; however, fixation on the coin significantly decreased after training (*p* < .01). Error bars represent mean ± standard deviation (SD)

**Table 2:** Fixation percentage (%) during the lift phase for each AOI and group (Mean ± SD)

AOI	Domi-BP	Domi-MP	Non-BP	Non-MP	*p*-value (ANOVA)
Coin	18.32 (6.30)	17.43 (6.29)	14.79 (7.60)	20.14 (3.82)	0.38 (n.s.)
Hand	9.37 (7.23) ^a^	24.38 (6.87) ^b^	8.19 (5.67) ^a^	28.68 (13.63) ^b^	< 0.001***
Jar	37.57 (14.14)	31.08 (13.75)	37.93 (10.26)	26.69 (5.89)	0.17 (n.s.)

Values represent mean ± standard deviation (*n* = 9 per group)

Groups sharing the same superscript letter are not significantly different (Tukey’s HSD, *p* < .05)

Superscripts indicate significant differences only between BP and MP groups

ANOVA revealed a significant group effect for Hand fixation (F(3, 32) = 10.93, *p* < .001, η² = 0.51), whereas Coin and Jar showed no significant differences

During the Lift phase, the fixation percentage directed toward the jar significantly increased from pre- to post-training in the Domi-BP group (F(2,16) = 10.378, *p* < .01, η² = 0.10) and the Non-BP group (F(2,16) = 4.912, *p* < .05, η² = 0.084), as revealed by simple main effects analysis following a significant Session × AOI interaction. In contrast, both MP groups showed no significant change in jar fixation, with gaze remaining primarily directed toward the hand and coin. Notably, in the Non-MP group, fixation time on the coin significantly decreased after training, representing the only change in gaze allocation observed within the MP groups.

Additionally, no significant main effect of hand dominance (dominant vs. non-dominant) was observed for any AOI.

To further clarify group-level differences, a one-way ANOVA was conducted for each AOI (Coin, Hand, and Jar) during the Lift phase across the four groups (Domi-BP, Domi-MP, Non-BP, and Non-MP). The analysis revealed a significant group effect for Hand fixation (F(3, 32) = 10.93, *p* < .001, η² = 0.51). Post-hoc comparisons indicated that both MP groups (Domi-MP and Non-MP) exhibited significantly higher fixation on the hand compared with BP groups, suggesting persistent visual monitoring during prosthetic control. No significant differences were observed for Coin or Jar fixation. These results are summarized in Table 2.

### Gaze behavior: blink rate (Fig. 9)

**Fig. 9:**
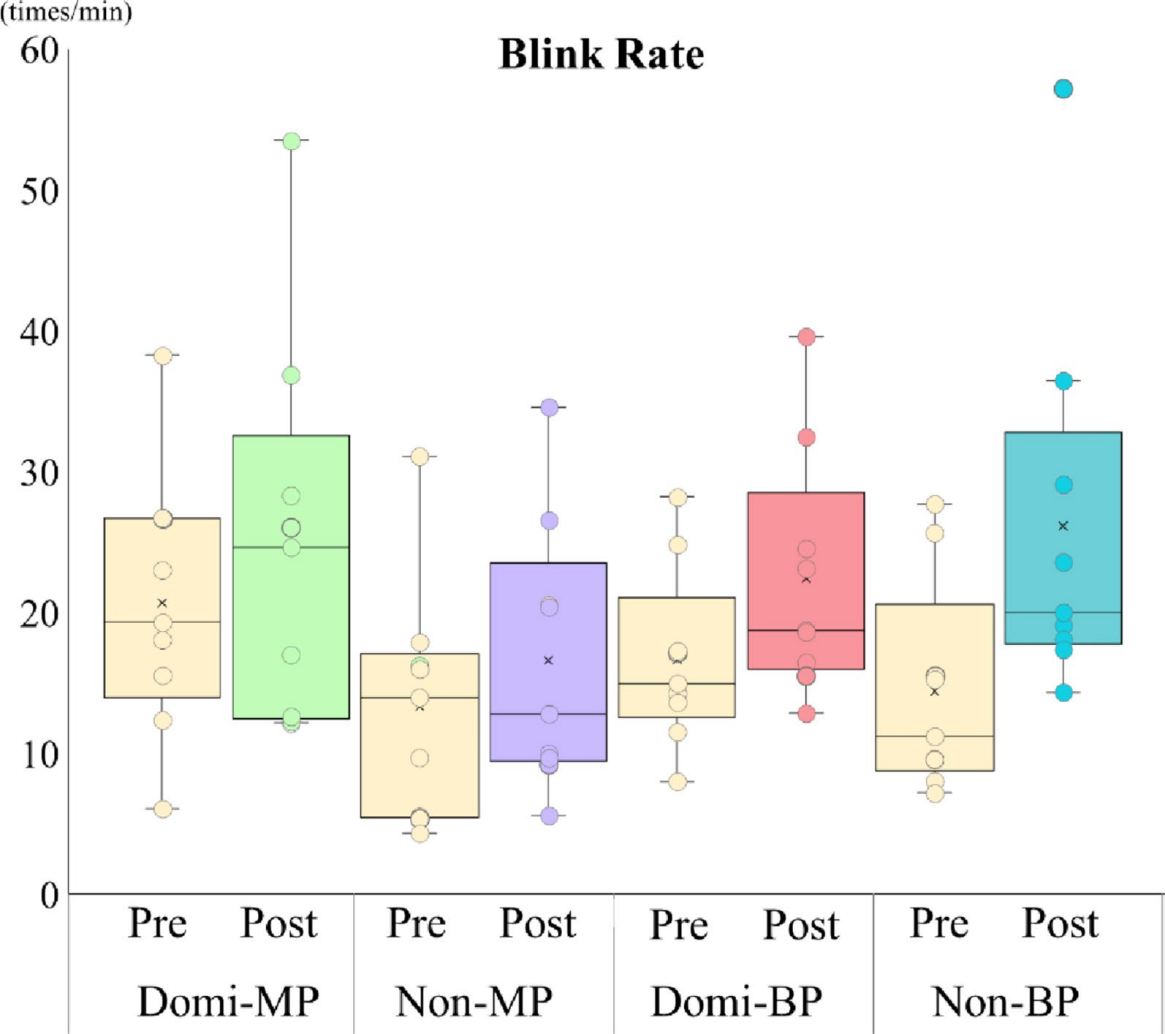
Blink rate before and after training. Blink rate did not significantly change across groups, suggesting stable cognitive load throughout the training period

No significant changes in blink rate were observed from pre- to post-training across all groups (main effect of Session: *p* > .05). No significant Session × Group interaction was found. These results suggest that overall cognitive load remained stable during the intervention.

### Gaze behavior and prosthesis ownership in individuals with upper limb amputation (Table 3; Fig. 10)

**Table 3:** Demographic characteristics and prosthesis use data of participants with upper limb amputation

ID	Sex	Age	Dominant hand	Amputation side and level	Causes of upper limb amputation	Prosthesis type	Duration of use (years)	Daily use* (h/day)
No.1	Female	72	Right	Right and Left forearm	Purpura fulminans	BP (Right and left), MP (Right)	BP (R/L): 1.8 MP (R): 1.0	BP (R/L): 10/13 MP (R): 3
No.2	Male	42	Right	Right forearm	Trauma	MP (Right)	5.5	16

*Daily use values were self-reported by participants as the average number of years and hours per day the prostheses were worn on typical weekdays

BP = body-powered prosthesis; MP = myoelectric prosthesis

**Fig. 10:**
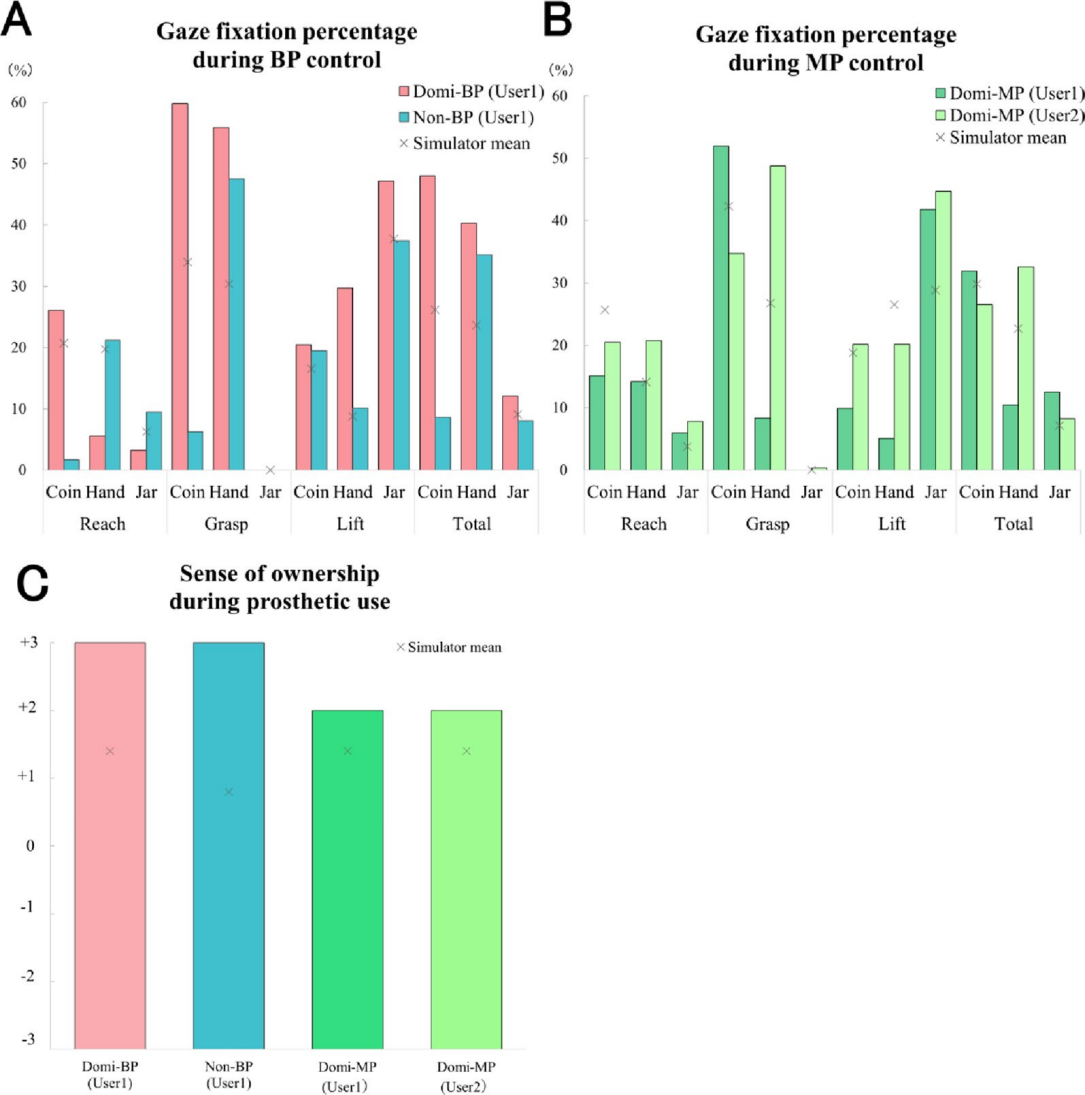
Gaze fixation percentage and sense of ownership during prosthetic use. (A) Gaze fixation percentage during Body-powered prosthesis (BP) control. (B) Gaze fixation percentage during Myoelectric prosthesis (MP) control. (C) Subjective sense of ownership ratings on a 7-point Likert scale (− 3 to + 3; higher values indicate stronger ownership). In the BP condition, high fixation percentages on the jar were observed during the Lift phase, consistent with the predictive gaze strategies reported in BP simulator users. In contrast, the MP condition showed similarly high fixation on the jar but differed from the simulator results, where hand-centered fixations were predominant. To facilitate visual comparison, each panel includes the type-specific simulator mean, calculated by averaging the dominant- and non-dominant-hand simulator groups within each prosthesis type (BP and MP). Both participants also reported high ownership scores (+ 3 and + 2), suggesting a strong sense of ownership developed through consistent, long-term prosthesis use

As a supplementary investigation, we collected data from two participants with upper limb amputation who routinely use prosthetic devices in daily life (Table 3) and asked them to perform the same task (Coins Task). Both participants performed the task using their own prosthetic devices (BP or MP), rather than the simulator, to ensure that the data reflect habitual control and long-term adaptation. User 1 used both BP and MP, whereas User 2 used only MP. Both users had relatively long histories of prosthesis use and high daily wear durations.

Regarding gaze behavior, the BP user demonstrated a high percentage of fixation on the jar during the Lift phase (Fig. 10A), indicating predictive gaze strategies consistent with those observed in the healthy BP group. Similarly, the participants with upper limb amputation who used MP also exhibited high jar fixation (Fig. 10B), revealing gaze patterns that contrasted with the hand- and coin-focused fixation observed in healthy MP users.

To facilitate direct visual comparison, Fig. 10 presents data from participants with upper limb amputation together with the combined mean fixation percentages of the simulator groups for each prosthesis type. Specifically, both Fig. 10A (BP condition) and Fig. 10B (MP condition) display data from participants with upper limb amputation alongside the type-specific simulator mean, calculated by averaging the dominant- and non-dominant-hand simulator groups within each prosthesis type.

In terms of subjective sense of ownership, both participants with upper limb amputation reported high scores (+ 2 to + 3) on the statement “I felt as if I was using my own hand,” suggesting a strong and stable sense of ownership that may have developed through long-term, daily prosthesis use (Fig. 10C). Figure 10C also integrates data from participants with upper limb amputation with the simulator group means, allowing intuitive comparison of subjective ownership levels between clinical and experimental participants.

## Discussion

This study aimed to clarify differences in gaze behavior, cognitive load, and sense of ownership during prosthetic control learning with BP and MP. The results showed a significant increase in gaze fixation on the jar during the Lift phase in the BP groups, whereas the MP groups continued to focus on the hand or target object after training. While all groups showed increased ownership scores post-training. No significant changes in blink rate—an index of cognitive load—were observed. Based on these findings, we provide the following detailed discussion.

### Differences in gaze strategies by prosthesis type

In the BP groups, increased fixation on the jar during the Lift phase indicated the development of predictive gaze—a strategy in which the gaze shifts to the next goal before the hand reaches it [6]. Predictive gaze supports smooth motor sequences by acquiring relevant visual information in advance, thereby promoting efficient visual attention [6]. This strategy can enhance task efficiency by increasing movement predictability and reducing search time [7].

Previous studies have shown clear distinctions in gaze behavior between natural and prosthetic hand use. Parr et al. [7] reported that able-bodied participants performing a coin transfer task exhibited earlier, predictive gaze shifts toward the next target when using their anatomical hand, whereas prosthesis use induced prolonged hand-centered fixations and delayed gaze disengagement.

These findings indicate that natural hand use involves a more anticipatory and efficient gaze strategy, which can serve as a reference for interpreting gaze behavior during prosthetic control.

Furthermore, the between-group comparison conducted during the Lift phase revealed that both MP groups exhibited significantly higher fixation percentages on the hand compared with BP groups (Table 2). This quantitative finding supports the interpretation that MP control relies more heavily on continuous visual monitoring, whereas BP control allows for more efficient and predictive gaze allocation toward the next target.

These results provide empirical evidence for distinct visuomotor strategies between prosthesis types, highlighting the need for training approaches that specifically address the excessive visual reliance observed in myoelectric control.

BP control involves direct body movement (scapular abduction and shoulder flexion) transmitted via a cable to operate the terminal device. This configuration permits the use of residual proprioceptive feedback (e.g., shoulder tension), potentially reducing reliance on visual input [1]. Because prosthetic control is aligned with internal bodily sensations, sensorimotor integration is improved, enabling successful task execution without the need for continuous visual monitoring of the hand.

Additionally, the voluntary-opening hook used in the simulated BP remained closed when the shoulder girdle was in a neutral position, minimizing the risk of coin drops. As a result, users did not need to visually monitor the hook during movement, facilitating gaze shifts toward the next target.

In contrast, the MP groups maintained gaze fixation on the hand or object, indicating sustained reliance on visual feedback. This reliance may stem from the limited sensory feedback in MP control [20, 21]. MP control involves potential time lags between voluntary muscle activation and mechanical response, as well as susceptibility to noise and misactivation, requiring users to visually confirm the outcome of each movement. These uncertainties may inhibit the development of predictive gaze strategies.

In addition, a significant reduction in gaze duration directed toward the coin was observed exclusively in the Non-MP group, which controlled the MP with the non-dominant hand. This may indicate a partial transition from object-focused visual attention to alternative gaze strategies, or a reallocation of cognitive resources. Given that the non-dominant hand is generally associated with lower motor control proficiency and requires greater attentional demands during novel tasks [10], it is plausible that participants in this group adopted compensatory visual strategies to manage the increased complexity and uncertainty of operating a myoelectric prosthesis—which inherently provides limited visual and sensory feedback. In contrast, no comparable change was found in the Domi-MP group, suggesting that handedness may modulate the development of gaze strategies during prosthetic control learning. These findings suggest that prosthesis type and structural characteristics influence gaze behavior, offering important implications for prosthetic design and training.

### Blink rate and cognitive load

Blinking serves as a physiological indicator of cognitive and mental load [8] and complements gaze behavior assessments. Blinks are typically suppressed during internal cognitive processing and are associated with focused attention and working memory. High cognitive engagement is often correlated with reduced blink frequency, reflecting task effort [8].

Although no significant changes in blink rate were observed, group-level trends by prosthesis type suggest differences in task demands and attention allocation. Simultaneous analysis of fixation and blink rate provides a more comprehensive view of cognitive resource distribution and may support the evaluation of training effectiveness and attentional burden.

### Hand-dominance effects

Previous studies have reported asymmetries between the dominant and non-dominant hands in terms of attentional resource allocation and predictive control strategies [10]. However, in the present study, no significant main effects of hand dominance were found for gaze fixation percentages, blink rate, or sense of ownership. This suggests that, during the early stages of prosthetic control learning, visuomotor strategies may be influenced more strongly by prosthesis type and task demands than by which hand is used.

In contrast, within the myoelectric prosthesis condition, some divergent patterns between dominant- and non-dominant-hand use were observed. Specifically, the Non-MP group showed a significant post-training reduction in fixation time directed toward the coin, whereas the Domi-MP group did not exhibit such a change. Although this effect did not appear as a main effect of handedness, it suggests that, in visually demanding control modes such as myoelectric operation, hand dominance may influence group-specific adaptation processes (simple effects).

Nevertheless, these differences were limited, and it is possible that hand-dominance effects did not fully emerge because participants were novices in prosthetic control. More complex tasks or longer-term training may reveal clearer dominance-related differences. Further research is needed to clarify when and how hand dominance influences visuomotor strategies during prosthetic learning.

### Changes in sense of ownership

Following training, all groups exhibited an increase in the sense of ownership; however, no significant differences were observed between groups. This finding suggests that repeated prosthesis use facilitated the sense of ownership of the device, regardless of prosthesis type. While embodiment is often used as a broader term encompassing both ownership and agency, the present findings specifically address the ownership component, reflecting the subjective experience that the prosthesis is part of the user’s own body. The sense of ownership is thought to arise from the integration of visual, motor, and somatosensory information [22], and the training tasks in this study were designed to facilitate such multisensory integration across all conditions.

Moreover, previous research has emphasized the importance of sensorimotor congruence in fostering the sense of ownership [23]. The voluntary motor experiences involved in operating the prosthesis may have contributed to the gradual incorporation of the device into the users’ body schema. Therefore, consistent and active engagement with the prosthesis through training appears to be crucial for enhancing ownership, regardless of prosthesis type.

Enhancing the sense of ownership is also important for supporting intrinsic motivation and sustained prosthesis use, as well as for facilitating the acquisition of operational skills [24]. Future interventions should aim to further enhance the sense of ownership by optimizing task design and incorporating sensory feedback mechanisms from the early stages of rehabilitation.

### Training implications based on gaze strategies

This study demonstrates that gaze strategies vary by prosthesis type, supporting the need for individualized training approaches. BP users may benefit from tasks that promote predictive gaze and facilitate attentional shifting. Gaze training can assist in reallocating visual attention more adaptively, thereby accelerating strategy acquisition [25].

Training that reinforces coordination between bodily movements and gaze—such as feedback on movement timing or previewing of visual targets—may further promote predictive gaze behavior. For MP users, strategies aimed at disengaging gaze from the prosthetic hand are crucial, given their tendency to visually monitor it.

Supplementary feedback, such as tactile or vibratory cues, may help mitigate visual dependence [26]. Real-time gaze feedback during training could also support metacognitive awareness and enhance refinement of gaze strategies [27].

Although predictive gaze allocation was more evident in the BP group, this did not necessarily translate into faster task execution. The absence of significant differences in task completion velocity suggests that predictive gaze reflects efficient visuomotor planning rather than purely mechanical performance advantages.

These individualized training methods may influence not only gaze behavior but also cognitive load, sense of ownership, and long-term prosthesis adherence. Future directions should include the development of adaptive training systems capable of analyzing gaze behavior in real time.

### Importance of multidimensional evaluation

This study employed a multimodal evaluation approach incorporating gaze behavior, blink rate, and the sense of ownership to assess prosthetic training outcomes. These indices effectively capture motor proficiency, cognitive workload, and subjective ownership-related experiences.

Schenk et al. [28] demonstrated the utility of simultaneously assessing gaze behavior, cognitive load, and embodiment-related measures in users of myoelectric prostheses.

Kilteni et al. [29] further emphasized that the sense of ownership, as a key component of embodiment, arises from the congruence of visual, motor, and tactile inputs, underscoring the importance of multisensory integration in prosthesis use.

Moreover, Siegle et al. [30] reported that blink rate is closely associated with cognitive load, supporting its use as a physiological indicator in prosthesis training.

Sobuh et al. [20] also advocated combining gaze metrics with subjective workload assessments to optimize training strategies for myoelectric prosthesis users.

Future research should aim to develop adaptive training systems incorporating real-time, multimodal feedback tailored to individual user needs. Intervention studies integrating gaze guidance and haptic feedback could further elucidate mechanisms of cognitive–sensorimotor integration and contribute to the development of more effective prosthetic training protocols.

### Integrated interpretation of multimodal findings

Although significant differences were observed only in gaze behavior between prosthesis types, the combined interpretation of gaze, blink rate, and sense of ownership provides complementary insights into prosthesis control.

Specifically, BP users demonstrated more predictive gaze allocation toward task-relevant targets, indicating efficient visuomotor planning. The absence of group differences in blink rate suggests comparable cognitive workload between BP and MP users, whereas the uniformly increased sense of ownership across groups reflects successful short-term embodiment acquisition through active engagement with the prosthesis.

Taken together, these findings suggest that gaze behavior serves as a sensitive and discriminative marker of prosthesis-specific control strategies, while blink rate and sense of ownership contribute to a broader understanding of attentional and perceptual adaptation during prosthesis learning.

This multimodal interpretation emphasizes the value of integrating behavioral and physiological measures to comprehensively evaluate cognitive and perceptual processes in prosthetic training.

Interpretation of gaze behavior and sense of ownership in participants with upper limb amputation.

The gaze behavior observed in the two participants with upper limb amputation provides preliminary insight into how long-term prosthesis use may influence visuomotor strategies. The BP user demonstrated a relatively high percentage of jar fixation during the Lift phase, a pattern broadly consistent with the predictive gaze tendencies shown by healthy BP simulator users. Although this observation is based on a single BP user, it may suggest that repeated and long-term BP use contributes to the development of object-centered, predictive gaze behavior.

In contrast, both participants with upper limb amputation who used MP exhibited high jar fixation, which differed from the strong hand-centered fixation commonly observed in healthy MP simulator users. This pattern may indicate that long-term use of a personalized MP prosthesis reduces the relative need for continuous visual monitoring of the hand, thereby allowing users to allocate more gaze resources to task-relevant objects. However, this interpretation should be viewed with caution due to the small sample size and the potential influence of individual differences.

Both participants with upper limb amputation also reported high sense of ownership scores, which may reflect the effects of consistent, long-term prosthesis use. The coexistence of relatively efficient gaze allocation and strong ownership is noteworthy, although the relationship between these factors cannot be established from the present data. Further research with larger and more diverse samples will be necessary to clarify the interplay between embodiment and visuomotor control.

Overall, the present findings suggest that the visuomotor strategies of experienced prosthesis users with upper limb amputation may differ from those used by novice simulator users, underscoring the importance of considering long-term adaptation and real-world experience when interpreting prosthesis control behavior. Nevertheless, given the limited sample size, these interpretations remain tentative and require further investigation.

## Limitations and future directions

This study primarily investigated short-term task familiarization rather than long-term prosthetic training. Therefore, the findings should be interpreted as reflecting early-stage adaptation rather than consolidated skill acquisition, which may limit generalization to clinical rehabilitation contexts.

First, participants were healthy individuals with no prior experience in prosthesis use, and their gaze behavior and sense of ownership may differ from those of individuals with upper limb amputation. Although healthy individuals can acquire simulated prosthesis control over a relatively short period, individuals with upper limb amputation may require extended durations to achieve a stable sense of ownership and psychological adaptation.

Second, this study focused on short-term task familiarization (pre–post training) rather than long-term prosthetic learning. Therefore, the observed improvements likely reflect task-specific adaptation rather than generalized skill acquisition. Future longitudinal studies are required to examine how gaze strategies, sense of ownership, and motor performance evolve over extended training periods.

Third, blink analysis was limited to average frequency and did not account for phase-specific timing. Future studies should investigate temporal blink dynamics and incorporate additional physiological indicators, such as pupil diameter, to better characterize cognitive load during prosthetic use.

Fourth, this study did not include a condition in which participants used their intact dominant or non-dominant hand. Future studies incorporating such a reference condition would enable a more direct comparison between natural hand use and prosthetic control, thereby clarifying how gaze strategies differ from typical eye–hand coordination patterns.

Despite these limitations, the present findings clarify differences in gaze strategies and cognitive load according to prosthesis type and provide useful insights for designing individualized training protocols. Future research should aim to develop gaze-guided training systems and real-time gaze feedback tools to facilitate prosthetic skill acquisition.

In addition, we supplemented our findings with gaze behavior and prosthesis ownership data from two participants with upper limb amputation (User 1: BP and MP user; User 2: MP user; see Fig. 10; Table 3). For the BP user, a high percentage of gaze fixation on the jar was observed during the Lift phase, demonstrating a predictive gaze strategy similar to that of the healthy BP simulator group. Similarly, the MP users also exhibited high jar fixation, contrasting with the hand-focused gaze patterns observed in healthy MP simulator users.

These findings suggest that long-term, high-frequency prosthesis use contributes to the development of proficient, predictive gaze strategies. Furthermore, both users reported high ownership scores, indicating that their prostheses were well integrated into their body representation and perceived as part of their own bodies.

Taken together, these data from participants with upper limb amputation not only support the validity of the simulator-based findings in healthy participants but also underscore the potential generalizability of gaze strategies and the sense of ownership to real-world clinical contexts. Future studies should include larger cohorts of participants with upper limb amputation and longitudinal designs to more thoroughly examine how accumulated prosthesis experience shapes gaze behavior and the development of the sense of ownership.

## Conclusions

This study clarified the effects of prosthesis type on gaze strategies and the sense of ownership. In the BP group, predictive gaze shifts were facilitated, whereas in the MP group, fixations on the hand or target object were maintained, indicating sustained reliance on visual feedback. Although no significant differences were found in blink rate, the multidimensional evaluation combining gaze behavior, blink rate, and ownership proved effective in understanding cognitive load and ownership-related experiences during prosthesis use. These findings underscore the importance of individualized training strategies tailored to prosthesis type and highlight the potential value of future intervention studies incorporating gaze-guided training and real-time feedback to enhance prosthesis control and sense of ownership.

## Data Availability

The datasets generated and/or analyzed during the current study are available from the corresponding author upon reasonable request.

## Abbreviations

BP: Body-powered prosthesis
MP: Myoelectric prosthesis
EMG: Electromyography
Domi-BP: Dominant-hand body-powered prosthesis group
Non-BP: Non-dominant-hand body-powered prosthesis group
Domi-MP: Dominant-hand myoelectric prosthesis group
Non-MP: Non-dominant-hand myoelectric prosthesis group
